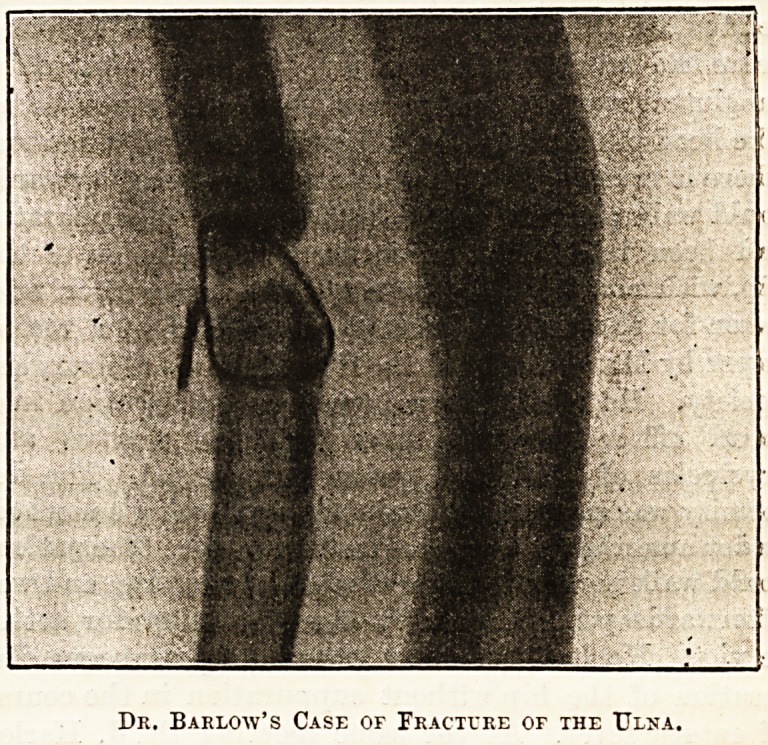# Progress in Surgery

**Published:** 1896-10-31

**Authors:** 


### Progress in Surgery.

### GENERAL SURGERY.

*(Continued from page 48.)*

In a discussion at the College of Physicians of Philadelphia on a case of fracture of the coronoid process of the ulna, described by Dr. Thos. F. Branson,[30] Dr. O. H. Allis remarked that the lower humeral articulation was so exact that a dislocation was out of the question, without, as a rule, extensive laceration of the lateral ligaments. In experimental work he had chiselled away little by little the coronoid process, making with each lessening of the coronoid an attempt to dislocate, but found it impossible, even after its complete removal, to dislocate the ulna backwards without extensive laceration of the lateral ligaments. When dislocation backwards occurred through leverage or twist, the violence expends itself chiefly upon the lateral ligaments, and the coronoid escapes, or, if injured, only the tip is broken off. Two skiagraphs of ununited fracture of the forearm have recently been reproduced. In the first instance, from a patient of M. Page,[31] of Newcastle, the precise nature of the injury to the forearm was at the time uncertain. The new photography immediately revealed the site and position of the fracture, and the usual treatment was at once employed. The second, given by Dr. J. Macintyre,[32] (see Fig.) from a patient of Dr. Barlow's, shows the position of the wire after operation for ununited fracture of the ulna. Mr. Sydney Rowland[33] mentions a third case in which non-union of both bones was supposed to have been due to impaction of soft tissue. The skiagraph here revealed unsuspected conditions, which will modify the further surgical proceedings, for it showed great displacement and overlapping of fragments to the extent of quite half an inch, without impaction of soft tissue. Skiagraphy as an aid to the diagnosis of epiphysial lesions of chilhood was the subject of a brief note by Mr. John Poland,[34] who figures and reports a very rare case of separation and displacement forwards of the epiphysial head of the second metacarpal bone. The skiagraph was taken by Mr. Rowland from a patient aged seventeen years, nearly three years after the accident. It confirmed absolutely the lesion which was diagnosed at the time. Mr. Poland believes the importance of obtaining skiagraphs of certain forms of recent complicated injuries to the epiphyses in children to be very great. At the lower end of the humerus, for example, there are but few anatomical specimens in

Dr. Barlow's Case of Fracture of the Ulna.

museums to guide the surgeon in making an exact diagnosis of the numerous and complicated lesions which, it cannot be doubted from daily experience, must exist during life. Could this knowledge be obtained by Röntgen's method it will, he believes, materially assist the surgeon in giving a decision as to immediate operative measures in elbow-joint injuries of children, and in removing some of the many instances of subsequent severe deformity and loss of function of joint and limb, now so little creditable to surgery. A case of dislocation of the finger under the care of Mr. F. Page[35] was also diagnosed by this method. The patient, a lad, had received an injury to the ring finger of his left hand fifteen months ago. When seen three months ago it was not possible to determine whether he had sustained a fracture or a dislocation. The skiagraph at once placed this beyond doubt.

*Lower Extremity.*—Two instances of unreduced dislocation of the hip into the obturator foramen have recently been shown at the Medical Society. In the first case, on account of the crippled condition, Mr. Johnson Smith[36] cut down and removed the head of the bone. The result was excellent so far as the hip was concerned, but it then became evident that there was extreme paralysis of the extensor muscles of the leg, causing a very faulty position of the foot. Whether this was present before the operation could not be ascertained. The second case came under the care of Mr. G. R. Turner many weeks after the accident, with the usual signs of dorsal dislocation, but crepitus of uncertain nature could be felt, so that it was possible that there was fracture as well as dislocation. He thought the only treatment under these circumstances was excision of the head of the bone. Dr. J. B. Roberts[37] describes a case of iliac dislocation complicated with fracture of the head and neck of the femur and of the edge of the acetabulum. It occurred in a man aged twenty-five years. More than a month after the accident arthrotomy was performed, after an unsuccessful attempt had been made at reduction, which was thought to have caused the fracture. Death ensued an hour after from cardiac embolism. Dislocation of the head of the femur consecutive to typhoid fever occurred in a case related by Dr. A. B. Hirsh.[38] A girl aged fourteen years had an attack of typhoid fever complicated by subgluteal abscess and distension of the acetabulum. The head of the femur had slipped down to the dorsum, where it remained. The patient five years afterwards could walk very well with a high heel, and all apparatus had been laid aside. Spontaneous dislocation of the hip, with some remarks on a class of cases often mistaken for rheumatism, formed the subject of a recent paper by Mr. Barwell[39] at the Royal Medical Chirurgical Society. He narrated an instance of dislocation in a naval officer which followed multiple abscess and erysipelas of the face, contracted abroad. The deformity was successfully reduced by Bigelow's method, under anæsthesia, with an excellent result. The patient could walk perfectly straight without limping, and was afterwards passed again by the Admiralty for active service. He had seen four cases of spontaneous dislocation of the hip without suppuration in the course of enteric fever. At the same meeting Dr. J. Harley referred to a case of spontaneous dislocation of the shoulder following gonorrhœal rheumatism in a young woman. It was easily reduced, the arm bound up to the side, and the elbow supported. The result was a good recovery. Such a case showed that surgeons should not too quickly interfere in cases of joint affection, which might get well of themselves. In some cases of pyæmia complete absorption of pus from joints might occur. Surgeon-Colonel Stevenson[40] mentions a case of fracture of the femur into the knee-joint, in which the advantage of the new photography, both as regards prognosis and treatment can hardly, as he says, be overrated. The fracture was situated three inches above the condyles, and was produced by indirect violence in a man aged thirty-three. Two skiagraphs were taken ten days afterwards by Mr. Rowland. The first, from side to side, showed the shortening and the partial separation of the fragments; and the second, from front to back, showed a line of splitting from the site of the fracture into the joint through the intercondyloid notch. Two interesting cases of separation of the lower epiphysis of the femur treated by operation are reported by Dr. C. McBurney.[41] In both the stripped up periosteum remained largely continuous to the epiphysis, and added to the difficulties of reduction. The numerous articles recently published and methods proposed sufficiently prove that the treatment of fractured patella is often difficult and unsatisfactory. Dr. J. D. Bryant[42] agrees with many other surgeons that the patella should not be wired simply because it is broken. The method he describes has nothing particularly novel, he says, but commends itself because the patient can be up and about his room in about ten days. Continuous extension is employed without confinement; but, as Dr. Abbe states, it is somewhat too elaborate for ordinary use. Bähr[43] asserts that muscular contraction alone will not cause fracture of a normal patella. Indirect transverse fracture, it is believed, is caused by sudden tension of the ligamentum patellæ whilst the knee is semiflexed. It is often associated with laceration of other soft parts about the knee-joint, and is followed by long-continued atrophy of the quadriceps and restricted flexion of the joint. These bad results are quite independent, he says, of any special mode of treatment. Mr. A. E. Barker's latest communication[44] on the subject dwells on the author's well-known method of permanent subcutaneous suture. He states that since 1894 he has employed it in every case of fresh fracture admitted to his wards. To get the best results it should be done as soon as possible after the injury, and the blood in the joint should be squeezed out through the punctures made into the joint, while massage is begun the day after operation, and continued for two weeks. Passive motion, too, should be commenced at an early date. Barker insists upon these details (not mentioned in his other papers) being carefully attended to, and draws attention to a needle specially designed to carry the thick wire, and also to metal bars through which are threaded the upper and lower ends of the wire placed vertically round the fragments, to enable the surgeon to draw them evenly together and twist them accurately. Isolated fractures of the head of the fibula are of rare occurrence. Dr. A. Trietze[45] reports a case, in addition to the twelve recorded by Dumollard in 1882. They occasionally occur from direct violence, but more often from muscular action. The usual treatment is to replace the fragments as well as possible, and place the leg in a flexed position until union occurs. The case reported is the first in which the fragments have been sutured, although the operation has been strongly recommended by Weir. An interesting example of deficient development of the lower end of the tibia after transverse fracture is reported by Mr. Gordon Brodie.[46] The patient, aged twenty-two, had sustained a fracture of the tibia about its middle twelve years previously. The lower end of the tibia was dwarfed, with an inward curve of its lower fourth, bringing the internal malleolus into strong relief, and causing the tibio-fibular mortise to be inclined slightly inwards, thus giving the foot a list inwards. There was no irregularity of the anterior border of tibia, but considerable hypertrophy of the lower end of the fibula, whose subcutaneous surface was marked by ridges. The foot assumed the position of talipes valgus. Dr. F. Bauer[47] reported to the Swedish Medical Society ten cases of the ambulant treatment of fractures of the lower extremity, viz., three fractures of both malleoli (one of them compound), one Dupuytren's fracture, one fracture at upper and middle third of tibia, one complicated fracture at middle of tibia, one fracture of middle of tibia and upper third of fibula, one fracture of patella, one fracture above condyles of femur, and one case of pes valgus, where osteotomy was performed on the tibia and fibula. In no case was it necessary to remove the plaster of Paris bandage on account of pain or pressure. In nearly every case the patient left the bed the day after the bandage was put on, and then continued moving about, at first with the aid of crutches, then with a cane, or with no extra support at all. The stiffness of the knee-joint and the atrophy of the muscles soon disappeared under proper treatment. To these cases Bauer has added eight others since treated successfully by the same method, viz., one supracondyloid fracture of the femur, one V fracture of the leg, one ordinary fracture of the leg, three supramalleolar fractures of tibia and fibula, and one Dupuytren's fracture. Dr. E. Martin[48] has tried the ambulatory treatment in a limited number of suitable cases, *i.e.*, of simple fracture of the leg seen early. In not a single instance has he had bad results. Dr. L. McLane Tiffany[49] gives a description of his treatment of compound fractures of the lower extremity under four headings: (1) Cleansing of the limb; (2) as to the strangulation which will follow the swelling; (3) bringing of the parts together; (4) sustaining the fragments by apparatus until definite union has occurred. He employs extensive shaving of the limb and use of antiseptics, and makes free the bones by incising the deep fascia to prevent extravasation of blood along the limb. The bones then come easily together. If there is much deformity, however, he wires them and leaves the wire in. He never closes the wound under any circumstances, but puts on a voluminous dressing of sterilised gauze, then cotton, and lastly plaster of Paris bandage. The limb is afterwards suspended from the ceiling for several weeks without the wound being inspected. A granulating wound is then found level with the skin. Sprains and their treatment form the subject of a paper by Dr. J. C. Biddle.[50] If it is difficult, he says, to make a diagnosis in sprain of the ankle, knee, wrist, elbow, and shoulder, it is infinitely more so in severe sprains of the vertebral column. In the treatment of sprains of the joints of the lower extremity, he employs rest (by splints), compression (by oakum, &c.), passive motion (on the third or fourth day), forcible manipulation, friction, and massage. Dr. A. Hoffer[51], of Würzburg, obtains excellent results in the treatment of sprains of the ankle joint from the use of a bandage, composed of strips of diachylon, by which the patient is enabled to walk without difficulty. This form of bandage was first introduced by Mr. E. Cotterell. If the patient is seen immediately after the accident, this bandage should be applied at once; if after the lapse of some time, and swelling has occurred, the limb is first placed in a raised position, and compression employed by means of a rubber bandage, the diachylon bandage not being applied until twenty-four hours later, when the swelling has considerably diminished.

[30] Annals of Surg., April. 1896, p. 44. [31] Lancet, April 25, 1896, p. 1,159. [32] Glasgow Med. Journ., April. 1896, p. 277. [33] B. M. J., April 25. 1896, p. 1,059. [34] B. M. J., Mar. 7, 1896, p. 620. [35] Ibid., p. 621. [36] B. M. J., Feb. 15, 1896, p. 404 [37] Annals of Surg., Feb., 1896, p. 207. [38] Ibid., p. 212. [39] Lancet. May 2, 1896, p. 1,222. [40] B. M. J., April 25, 1896, p. 1,059. [41] Annals of Surg., April, 1896, p. 501. [42] Med. Record N. Y., April 4, 1896, p. 472. [43] Archiv. für Unfallheilkunde Bd. I. Heft. 1. and Epit. B. M. J., May 2, 1896 [44] B. M. J., April 18, 1896, p. 963. [45] Archiv. für Klin. Chir. Bd. XLIX., Heft., 2, and Annals of Surg., Feb., 1896, p. 242. [46] Lancet, May 2, 1896, p. 1,226. [47] Annals of Surg., Feb., 1896, p. 240. [48] Annals of Surg., April, 1896, p. 462. [49] Ibid., p. 449 [50] Therapeut. Gaz., April 15, 1896, p. 217. [51] Med. Week, April 3, 1896, p. 167.

## Figures and Tables

**Figure f1:**